# Solid Lipid Nanoparticles for Duodenum Targeted Oral Delivery of Tilmicosin

**DOI:** 10.3390/pharmaceutics12080731

**Published:** 2020-08-04

**Authors:** Kaixiang Zhou, Yuanyuan Yan, Dongmei Chen, Lingli Huang, Chao Li, Kuiyu Meng, Shuge Wang, Samah Attia Algharib, Zonghui Yuan, Shuyu Xie

**Affiliations:** 1 National Reference Laboratory of Veterinary Drug Residues (H.Z.A.U.) and MAO Key Laboratory for Detection of Veterinary Drug Residues, Wuhan 430070, China; flyingkai@webmail.hzau.edu.cn (K.Z.); yuanyuanyan@webmail.hzau.edu.cn (Y.Y.); chaoli@webmail.hzau.edu.cn (C.L.); mengky@mail.hzau.edu.cn (K.M.); shugewang@webmail.hzau.edu.cn (S.W.); samahalgharib@163.com (S.A.A.); 2 MOA Laboratory for Risk Assessment of Quality and Safety of Livestock and Poultry Products, Huazhong Agricultural University, Wuhan 430070, China; chendongmei@mail.hzau.edu.cn (D.C.); huanglingli@mail.hzau.edu.cn (L.H.); yuan5802@mail.hzau.edu.cn (Z.Y.); 3 Department of Clinical Pathology, Faculty of Veterinary Medicine, Benha University, Moshtohor, Toukh 13736, Egypt

**Keywords:** antimicrobial-resistance, tilmicosin enteric granules, P-glycoprotein, duodenum-targeted release, oral absorption, reduce consumption

## Abstract

Developing a targeted oral delivery system to improve the efficacy of veterinary antibiotics and reduce their consumption and environmental risks is urgent. To achieve the duodenum-targeted release of tilmicosin, the enteric granule containing tilmicosin-loaded solid lipid nanoparticles (TIL-SLNs) was prepared based on its absorption site and transport characteristics. The in vitro release, release mechanisms, stability, palatability, and pharmacokinetics of the optimum enteric granules were studied. The intestine perfusion indicated that the main absorption site of tilmicosin was shifted to duodenum from ileum by TIL-SLNs, while, the absorption of TIL-SLNs in the duodenum was hindered by P-glycoprotein (P-gp). In contrast with TIL-SLNs, the TIL-SLNs could be more effectively delivered to the duodenum in intact form after enteric coating. Its effective permeability coefficient was enhanced when P-gp inhibitors were added. Compared to commercial premix, although the TIL-SLNs did not improve the oral absorption of tilmicosin, the time to reach peak concentration (T_max_) was obviously shortened. After the enteric coating of the granules containing SLNs and P-gp inhibitor of polysorbate-80, the oral absorption of tilmicosin was improved 2.72 fold, and the T_max_ was shortened by 2 h. The combination of duodenum-targeted release and P-gp inhibitors was an effective method to improve the oral absorption of tilmicosin.

## 1. Introduction

Veterinary antibiotics (VAs) are indispensable for controlling and preventing microbial infections in livestock. According to the literature [1], 80% of antibiotics sold in the USA in 2009 were used on farms. Simultaneously, the overexposure of VAs to animals and the environment caused by their abuse and heavy usage in veterinary clinics will accelerate the emergence of antimicrobial resistance (AMR) which threatens public health [2]. Currently, approximately 700,000 people die each year because of AMR; it will reach 10 million people by 2050 [3]. For animal farming, due to the fact of higher compliance and lower professional requirements than other routes, the oral route is the most preferred of all of the administration routes [4]. According to the literature, approximately 80% of drugs were administrated orally [5]. However, the unnecessary metabolic waste and poor oral absorption are usually due to the challenges of poor solubility, poor permeability, gastrointestinal degradation, the first pass effect, and release in non-major absorption sites [6], thus causing heavy usage of VAs. Meanwhile, with the poor adsorption of VAs in the gut of animals, the majority are excreted unchanged in feces [7]. There is no doubt that this will cause serious environmental risks. Innovation in veterinary pharmaceuticals is required for enhancing its absorption and efficacy, in addition to decreasing dosages and environmental discharge (e.g., manure). Therefore, developing effective oral antimicrobials products to reduce the consumption of VAs is necessary.

Due to the fact of their small size and large surface area, solid lipid nanoparticles (SLNs) are often used to increase the sustained-release performance and transmembrane ability, and thus, improve the peak concentration (C_max_) and oral absorption of drugs [8]. For example, SLNs significantly enhanced the C_max_ of geniposide [9]. It was reported that SLNs could improve the oral bioavailability of camptothecin and enrofloxacin [10,11]. It should be noted that SLNs has emerged as an effective carrier to reduce the first pass effect of drugs via protecting drugs from degradation by lipid materials and change internalization pathways after their being nanosized [12]. Therefore, SLNs are considered to be an effective way to improve the oral absorption and in vivo disposition of tilmicosin.

Different SLNs have different absorption sites in intestines and various transport routes involved in the transportation of lipid-based nanoparticles by intestine cells due to the difference in lipid materials and physicochemical properties [13]. Li et al. [14] proved that quercetin-loaded SLNs were mainly absorbed in the ileum and colon. It was reported that the order of the main absorption sites of insulin-loaded SLNs was ileum > jejunum > duodenum [15]. However, considering the number of villi and microvilli, the duodenum and jejunum are the main absorption sites of drugs [16], and the duodenum target is undoubtedly beneficial for the complete absorption of drugs as it is the first section of the intestines. Zhang et al. [17] reported that the absorption of candesartan cilexetil (CC) in gastrointestinal tract was only 2.8%, and SLNs increased the oral absorption of CC by lymphangion. Since the doxorubicin hydrochloride enoxaparin sodium-poly (lactic-co-glycolic acid) hybrid nanoparticles could escape P-gp mediated efflux, the oral absorption of doxorubicin hydrochloride was improved [18]. Therefore, the uptake mechanism of SLNs also plays a key role in the design of the SLNs with high oral absorption. Meanwhile, excipients and some emulsifiers (e.g., sucrose monolaurate and polysorbate-80) showed the potential for inhibiting the activity of P-gp in intestinal cells and, thus, enhanced the oral absorption of drugs [19], which suggests the combination of nanoparticles with P-gp inhibitors will be a potential way to further improve the oral absorption of nanoparticles.

However, the destruction of gastric juice to SLNs cannot be ignored, thus, resulting in a large number of premature releases of the drug before reaching the absorption sites. Our previous research indicated that the enrofloxacin-loaded stearic acid SLNs (ENR-SLNs) showed a massive release in the simulated gastric juice (SGF) [20]. The burst release of SLNs in gastric juice hinders its application as an efficient oral delivery system. To avoid the burst release of SLNs intragastric, intra-duodenal administration was adopted for its delivery [21,22,23], while it is not an appropriate method for administering drugs to animals repeatedly in veterinary clinics. After coated by enteric materials, the burst release of ENR-SLNs intragastric was obviously reduced [20]. Therefore, SLNs with enteric coatings may be effective for duodenum-targeted release to achieve the maximum absorption of their payload drugs. To prevent the tilmicosin-loaded solid lipid nanoparticles (TIL-SLNs) from being degraded by gastric fluid, thus achieving its duodenum-targeted delivery, the enteric coating based on the TIL-SLNs was adopted.

Tilmicosin is a new semisynthetic macrolide antibiotic that is semisynthetic from the hydrolysate of tylosin. Because of the low inhibitory concentration [24], extensive distribution volume [25], and rapid accumulation in deep tissue, tilmicosin was widely applied in veterinary clinics including for treatment of bacterial and *Mycoplasma* infections, especially for intracellular bacterial infections. Due to the cardiovascular toxicity of tilmicosin injection [26,27], tilmicosin was always recommended for oral administration. However, because of the incomplete oral absorption, it often shows varied oral bioavailability in different animals. Meanwhile, tilmicosin belongs to concentration-dependent antibiotics [28] with a long post effect [29]. Therefore, the higher C_max_ and longer-lasting high plasma concentrations were preferred. Because of these, a new oral drug delivery system that could improve the C_max_ and sustained-release performance of tilmicosin is needed. Thus, tilmicosin was selected as a model drug for developing the duodenum-targeted release delivery systems.

In order to design a kind of effective duodenum-targeted release of SLNs with the help of enteric granules, the absorption site and transportation characteristics of the developed TIL-SLNs were firstly studied by the intestinal perfusion in situ model and combined the transportation inhibitors to achieve the maximum absorption in the duodenum. Subsequently, the enteric granules which used the optimum TIL-SLNs as the inner core were prepared to reduce the destruction of SLNs by gastric juice and to ensure that the TIL-SLNs reached duodenum in the intact state. The characteristics, release mechanisms, palatability, stability, and oral absorption of the enteric granules were studied in detailed. This manuscript will provide the theoretical directions for the design of other antibiotic products with high oral absorption, thus reduce the consumption and improve the efficacy of VAs and decrease the AMR risks and environmental pollution caused by heavy usage of VAs.

## 2. Materials and Methods

### 2.1. Chemicals

Tilmicosin standard (content: 80.7%) was purchased from Ehrenstorfer (Augsburg, Germany). Native tilmicosin (content: >98%) was brought from QILU Pharmaceutical (Neimenggu, China). Tilmicosin premix was provided by Hvsen Biotech Co., Ltd. (Wuhan, China). Tilmicosin oral solution (content: 10%) was provided by Guangdong Wens Co., Ltd. (Guangdong, China). Indomethacin, verapamil, and EDTA-2Na were provided by Source Biotechnology Co., Ltd. (Shanghai, China). Poloxamer 188 (MW: 102.133, hydrophilic–lipophilic balance: 16.0), polyvinyl alcohol (PVA, MW: 30,000–70,000), polyacryl resin II (PR II, soluble pH > 6.0, equivalent to pig duodenum pH) were obtained from CHINEWAY (Shanghai, China). Pepsin (1:10,000) sucrose, starch, and carnauba wax were provided by Aladdin (Shanghai, China). Poly-vinyl pyrrolidone (PVP_K30_, MW: 111.144), sodium carboxymethyl cellulose (CMCC-Na), NaCl, MgCl_2_, CaCl_2_, KCl, NaH_2_PO_4_, and glucose were obtained from Sinopharm Group Chemical Reagent Co., Ltd. (Shanghai, China).

### 2.2. Animals

Sprague–Dawley rats (300 ± 15 g) were supplied by the Experiment Animal Center of Huazhong agricultural university (HAZU) (Wuhan, China). The rats were kept under standard conditions and with free access to food and water. Twenty-four clinically healthy three-way hybrid pigs (20–25 kg) were provided by Jinling pig farm (Wuhan, China). The pigs were fed at laboratory animal rooms at the National Reference Laboratory of Veterinary Drug Residues (HZAU). They were fed drug-free feed and water for seven days. The environment was kept at a suitable relative humidity (45–65%) and temperature (18–25 °C), respectively. All the experimental protocols were approved by the Institutional Animal Care and Use Committee at Huazhong Agricultural University (Approval number: HZAURA-2018-008, HZAUSW-2018-010, April 2019) and followed the guidelines of Hubei Science and Technology.

### 2.3. Preparation of TIL-SLNs Suspensions

The TIL-SLNs were prepared by a hot melt with an ultrasonic emulsification method, as described previously [30]. Briefly, 1 g tilmicosin was dissolved in the 2 g melted carnauba wax under stirring. After completely dissolving, boiling emulsifier (PVA, PVP, or poloxamer 188) water solution (water phase) was quickly mixed with the lipid solution (oil phase) under constant stirring to form a primary emulsion. Then primary emulsion was sonicated (probes diameter 1.2 cm, 90% power) (VCX750, Lueshen Instrument Equipment) for 5 min to obtain hot O/W nanosuspension. The hot O/W nanosuspension was cooled in an ice bath for 20 min to form TIL-SLNs. The optimal emulsifier type, aqueous phase concentration, and aqueous phase volume were evaluated by the orthogonal experiment (Table 1).

### 2.4. Characterization of TIL-SLNs

The shape of TIL-SLNs was observed by scanning electron microscope (SEM) (S-4800; Hitachi Limited Ltd., Tokyo, Japan). The size, polydispersity (PDI), and zeta potential of the TIL-SLNs were determined by Zetasizer ZX3600 (Malvern Instruments, Malvern, UK). The thermal analysis of TIL-SLNs was performed by differential scanning calorimeters (DSC, DSC200PC, NETZSCH, Selb, Germany) which was reported in our previous work [30]. The methods for determining the loading capacity (LC) and encapsulation efficiency (EE) have been clearly introduced in our previous reports [20,30]. The LC and EE were calculated as follows.

LC (%) = (Weight of tilmicosin in SLNs)/(Weight of SLNs) × 100%

EE (%) = (Weight of tilmicosin in SLNs)/(Weight of tilmicosin added) × 100%

### 2.5. Single-Pass Intestine Perfusion in Situ

**Figure 1:**
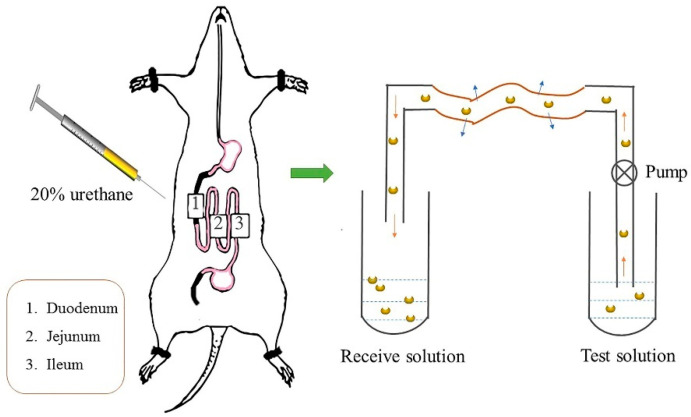
The schematic overview of the in situ intestine perfusion model.

To determine the main absorption site and transport characteristics of TIL-SLNs in the gastrointestinal tract, the rat single-pass intestine perfusion method was adopted. The method of single-pass intestine perfusion has been introduced in other papers [31,32,33,34,35]. Briefly, before the experiment rats were fasted for 12 h and free to drink water. During intestine perfusion, rats were anesthetized by 20% urethane (0.01 mL·g^−1^, intraperitoneal injection) and fixed on the operating table. The abdominal cavity was opened approximately 4 cm. Then, the ileum, jejunum, and duodenum were carefully separated and silicone tubes were inserted. The contents of the intestines were gently washed with saline (pre-warmed at 37 °C). After balancing with K-R (Krebs–Ringer buffer) solution (pre-warmed at 37 °C) for 20 min, the intestine was filled by the tilmicosin solution or TIL-SLNs suspension quickly (flow rate: 2 mL/min). Then, different tilmicosin solutions or TIL-SLNs were used for single-pass perfusion at 37 °C for 45 min at a flow rate of 0.2 mL/min. The perfused solution was collected and weighed every 15 min and repeat three times (Figure 1) [36]. After, the rats were sacrificed, and the various intestine sections were cut off to measure the length and radius. During the whole experiment, the wounds in the abdomen were covered by a cotton wool soaked with physiological saline buffer at 37 °C for moisturizing. The effects of various concentrations (0.06, 0.125, and 0.5 mg/mL, calculated by tilmicosin), gastrointestinal segments (duodenum, jejunum, ileum) and the adjust agents of transportation including EDTA (tight junction regulator 17 mmol·L^−1^), indomethacin (MRP2 inhibitor, 200 μmol L^−1^), and verapamil hydrochloride, polysorbate-80 (P-gp inhibitor) on the effective permeability coefficient (Papp) and absorption rate constant (Ka) of tilmicosin solution or TIL-SLNs were studied. The Papp and Ka were calculated using the following formulas:

$$Ka=\left(1-\frac{C_{out}}{C_{in}}\times \frac{V_{out}}{V_{in}}\right)\times \frac{v}{\pi r^{2}L}\;Papp=\frac{-\nu \times \ln\left(\frac{C_{out}}{C_{in}}\times \frac{V_{out}}{V_{in}}\right)}{2\pi rL}$$

where *ν* is the flow rate of perfusion (mL/min); *C_in_* and *C_out_* are the drug concentrations (mg/mL) in the perfused inlet and outlet, respectively; *V_in_* and *V_out_* are the volumes of the perfusion solution (mL) entering and leaving the intestine, respectively; *L* and *r* are the length (cm) and radius (cm) of the perfused intestinal segment, respectively [31].

### 2.6. Preparation and Characteristics of Enteric Granules Used TIL-SLNs as Kernel

To control the TIL-SLNs to be reached and released in its absorption site, the tilmicosin enteric granules contained the optimum TIL-SLNs was prepared. The preparation method of enteric granules was introduced in our previous work [20]. Briefly, the TIL-SLNs which contained 5% sucrose (*W/V*) was frozen dry for 48 h (Freeze Dry System; Labconco, America) to obtain the dry powder of TIL-SLNs. Then, the sucrose, starch, starch paste, and 1% (*V/W*) polysorbate-80 were respectively mixed with TIL-SLNs powder to prepare the suitable soft material. Then, the soft material was granulated by granulator (YK-160, Changzhou Lihao granulating drying equipment CO., LTD. Changzhou, China, screen mesh: 1 mm, two cycles). The prepared wet granules were put into the dry oven (GMP-O, Changzhou Lihao Granulating Drying Equipment Factory, Changzhou, China) (40 °C, 24 h) to remove extra water. Finally, the dried granules were coated with 3% (*W/V*) PR II ethanol solution using coating pan (BY-600, Zhong Nan Pharmaceutical Machinery factory, Changsha, China). After coating, the tilmicosin enteric granules containing TIL-SLNs were obtained and the appearance of the outer enteric coating membrane was observed by SEM.

### 2.7. In Vitro Release and Release Mechanism

The in vitro release performance of enteric granules and TIL-SLNs were studied in simulated gastric fluid (SGF, pH = 2.0) and simulated intestine fluid (SIF, pH = 6.8) by dissolution tester (ZRS-12S, Tianjin Xuyang Equipment Co., Ltd., Tianjin, China). Briefly, 500 mg enteric granules or 1 mL TIL-SLNs at the same amount of 50 mg within dialysis bags (MW: 20,000) was added in a dissolution cup containing 1000 mL buffer (38.5 °C, 75 rpm).5 mL sample was collected from the dissolution cup at fixed time points and filtered by 0.25 μm filter. Then 5 mL fresh SGF or SIF was added after sampling to keep a constant volume. The experiments were carried out in triplicate. The drug concentration was determined by HPLC. The in vitro release profiles were fitted by GraphPad Prism (version 7.0, GraphPad Software Inc., La Jolla, CA). Additionally, the enteric granules before and after release in SGF and SIF were observed by SEM to detect the completeness of the coating membrane, thus illustrate the in vitro release mechanism of enteric granules. Meanwhile, to prove the intact TIL-SLNs could be released in the duodenum, the enteric granules after incubation with SIF medium for 30 min was taken out and filtered by 0.65 μm filter and observed by SEM.

### 2.8. Stability of Enteric Granules

The storage stability of the tilmicosin enteric granules was evaluated by influencing factor experiments, including high humidity, high temperature, and strong light (ICH Topic Q1A (R2)). The 2.0 g enteric granules were placed in a container and then put in the environment of 40 °C (high-temperature test), 25 °C, 90% ± 5% (humidity test) or 4500 lux ± 500 lux (high light) for 10 days. The change in the appearance, drug content, granularity, in vitro dissolution, the completeness of inner TIL-SLNs and coating membrane were assessed on the 0th, 5th, and 10th day. To verify the completeness of inner TIL-SLNs, the enteric granules under the above conditions were ground on the 10th day by mortar and the powder was resuspended in water. Then the mixture solution was filtered by 0.65 um filter and observed by SEM to verify the completeness of inner TIL-SLNs.

### 2.9. Pharmacokinetics of Enteric Granules

To verify the assumption that the duodenum-targeted release of TIL-SLNs could improve the oral absorption, the pharmacokinetics of enteric granules in pigs was performed. Twelve healthy pigs before the experiment were randomly divided into 2 groups (commercial tilmicosin premix group and prepared enteric granules group) with 6 pigs in each group and kept for 7 days. After adaption period, tilmicosin enteric granules (content: 10%), and tilmicosin premix (Husen Biotech, Wuhan, China, content: 20%) were respectively re-suspended in 20 mL CMCC-Na and administered to the pigs by an intragastric route at a dose of 10 mg/kg b.w. After administration, 3 mL of blood was collected at 0.25, 0.5, 0.75, 1, 2, 4, 6, 8, 12, 24, 36, 48, 72, and 96 h, respectively. The blood sample was placed in a centrifuge tube containing heparin sodium and then centrifuged for 10 min at 4000 rpm to separate the plasma. After pretreatment, the drug concentration was detected by HPLC. Pharmacokinetic parameters were calculated by WinNonlin software (version 6.4; Pharsight Corporation, Mountain View, CA, USA).

### 2.10. Quantitative Measurement of Tilmicosin

The pre-treatment methods of blood samples and chromatographic conditions were introduced in our previous papers [20,30]. Briefly, 0.8 mL of plasma was added into a 10 mL tube containing 1.2 mL acetonitrile. The plasma and acetonitrile were thoroughly mixed under vortex for 2 min to precipitate protein and then centrifuged at 12,000 rpm for 20 min to take the supernatant. The supernatant was dried in nitrogen and reconstituted with an 800 μL mobile phase. Then, the liquid was filtered through a 0.22 μm filter and detected by HPLC (Waters 2695, Waters Corp., USA). The chromatographic conditions: column: (C_18_, 250 mm × 4.6 mm × 5 μm); detection wavelength: 287 nm; column temperature: 25 °C; mobile phase: water (phase A), acetonitrile (phase B), methanol (phase C), and 0.2 M ammonium formate (phase D, pH = 5.0) with the ratio of 32:24:24:20; flow rate: 1.00 mL/min; and injection volume: 10 μL. The content of tilmicosin was determined using a standard curve. The linear range of tilmicosin was from 0.05 to 4 μg/mL (*R*^2^ = 0.9999). The limits of detection and quantitation were 0.02 and 0.05 μg/mL, respectively. The RSD had a of precision was less than 2%, and the recovery rates of three different added concentrations were 95.1–101.4%.

### 2.11. Palatability Experiment

For the successful development of oral preparations, the acceptable palatability is important. Therefore, the palatability of various tilmicosin preparations was studied by feeding experiment of pig [20]. 

Data are presented as mean ± standard deviation (SD) and were analyzed by the SPSS software (version 20, IBM, New York, NY, USA). Statistical significance was defined as a *p*-value of 0.05 by the one-way ANOVA.

## 3. Results and Discussions

### 3.1. Optimization of TIL-SLNs

The saturation solubility of tilmicosin in hydrogenated castor oil and carnauba wax were 25% and 50% (100 °C, 30 min), respectively [30]. Due to the more significant solubility for tilmicosin, the carnauba wax was selected as the lipid matrix. When PVA was used as an emulsifier, the size of TIL-SLNs was within 1 um, and the size of using PVP or poloxamer 188 as an emulsifier was above 1 um. Both the PDI of the SLNs prepared by PVP and poloxamer 188 were >0.5, but the SLNs prepared with PVA were <0.5 (Table 2). Considering the industrial production and clinical application of tilmicosin, the high LC of TIL-SLNs was required. Therefore, the optimal TIL-SLNs were 25 mL, 3% PVA per 1 g tilmicosin, and 2 g carnauba wax.

One of the reasons why the LC was lower when using PVP and poloxamer 188 as an emulsifier, may be due to the solubilities of tilmicosin in PVP and poloxamer 188 were larger than that of in PVA. Furthermore, poloxamer 188 is always used to prepare the solid dispersion for improving the solubility of insoluble drugs [37,38]. Because of the low melting point of poloxamer 188, some tilmicosin may form a soluble solid dispersion with poloxamer 188 in the hot water thus leading to the low LC.

### 3.2. Physicochemical Properties of TIL-SLNs

**Figure 2:**
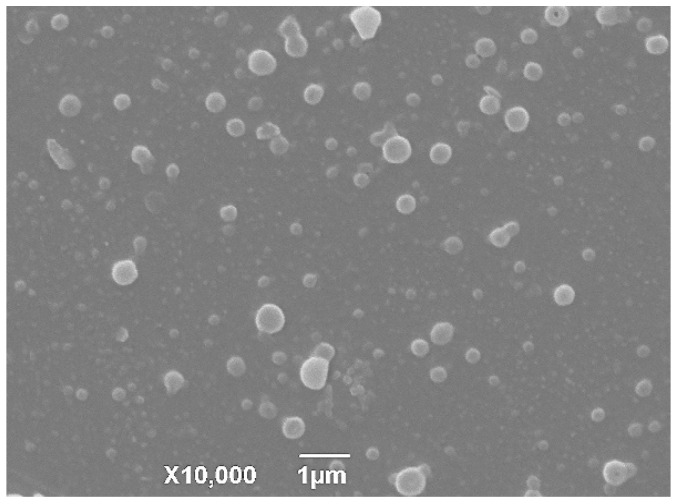
The SEM imaging of TIL-SLNs (tilmicosin-loaded solid lipid nanoparticles). The TIL-SLNs showed a spherical shape with a relatively narrow polydisperse.

The SEM imaging of TIL-SLNS showed that the TIL-SLNs was a spherical shape with a relatively narrow polydisperse (Figure 2). The mean sizes, PDI, and zeta potential of the TIL-SLNs were 368.3 ± 5.4 nm, 0.426 ± 0.025, and −12.0 ± 0.05 mv, respectively. The LC and EE of the SLNs were 21.04 ± 0.56% and 64.20 ± 2.35%, respectively. The LC data agreed with the conclusion of orthogonal design (Sample 4). The thermal analysis imaging of the TIL-SLNs showed that the melting point of carnauba wax and pure tilmicosin was 83.8 °C and 122.2 °C, respectively. Meanwhile, no tilmicosin peak was found in TIL-SLNs thermogram, suggesting the form of tilmicosin in the TIL-SLNs was disordered-crystalline phase (Supplementary Materials Figure S1) [30].

### 3.3. Absorption Site and Transport Characteristics of TIL-SLNs

**Figure 3:**
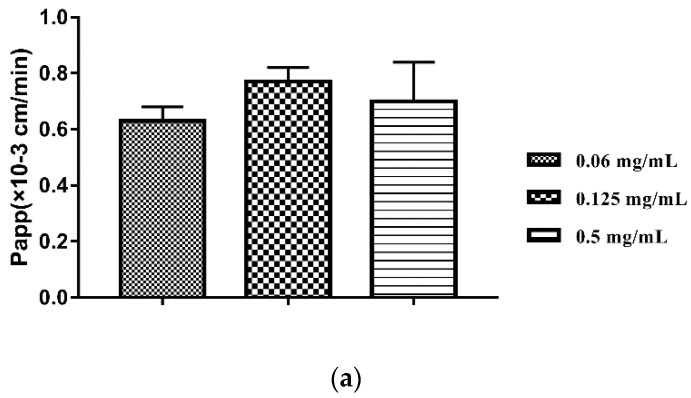

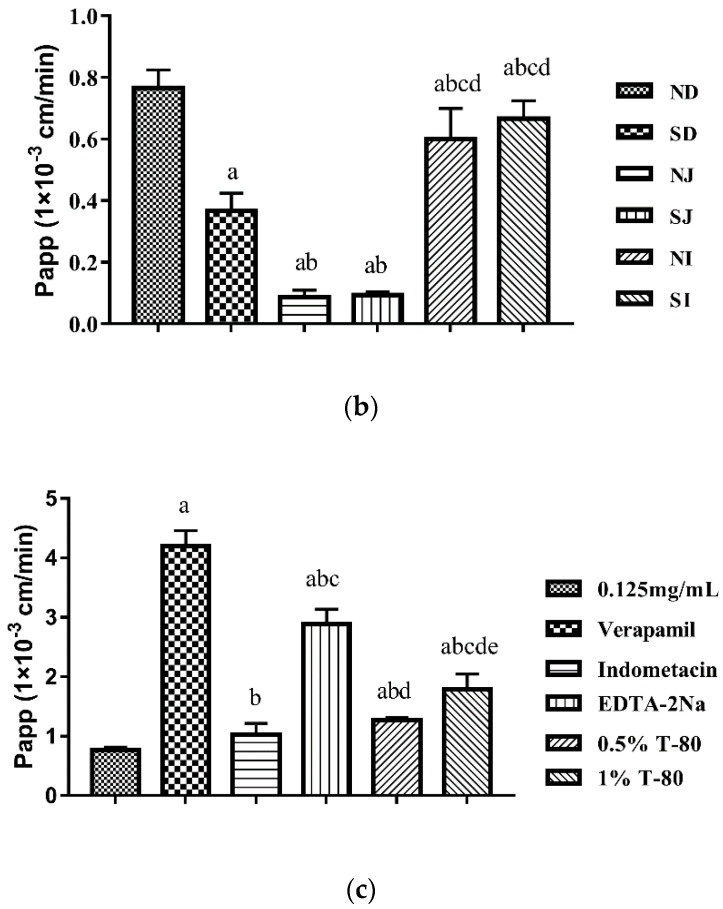
The influences of concentration (**a**), intestinal sections (**b**), and inhibitors (**c**) on the Papp of TIL-SLNs or tilmicosin solution. The absorption site of tilmicosin was shifted from ileum to duodenum via TIL-SLNs. The absorption efficiency of TIL-SLNs was hindered by P-gp. Note: (**a**), the Papp of various concentration of TIL-SLNs; (**b**), the Papp of TIL-SLNs in various intestine sections; ND: TIL-SLNs duodenum; SD: solution duodenum; NJ: TIL-SLNs jejunum; SJ: solution jejunum; NI: TIL-SLNs ileum; SI: solution ileum. ^a^ Statistically significant from TIL-SLNs duodenum; ^b^ Statistically significant from solution duodenum; ^c^ Statistically significant from TIL-SLNs jejunum; ^d^ Statistically significant from solution jejunum; (**c**), T-80: polysorbate-80, ^a^ Statistically significant from TIL-SLNs (0.125 mg/mL); ^b^ Statistically significant from verapamil; ^c^ Statistically significant from indomethacin; ^d^ Statistically significant from EDTA-2Na; ^e^ Statistically significant from 0.5% T-80. The statistical difference was analyzed by one-way analysis of variance at *p* < 0.05.

First of all, we used three concentration gradients (0.06, 0.125, and 0.5 mg/mL) to screen the optimal perfusion concentration. With the increasing of the concentration of perfusion solution, the Papp and Ka of various concentrations showed no obvious change (Supplementary Materials Table S1, Figure 3a). This reminded that the transport of TIL-SLNs was not concentration-dependent in order to obtain the data with a significant difference, the concentration with the largest Papp (0.125 mg/mL) was used to do the perfusion researches. The Papp and K a of various intestine sections indicated that the main absorption site of tilmicosin solution was ileum and the Papp in duodenum was larger than jejunum (Supplementary Materials Table S2, Figure 3b, *p* < 0.05). Compared with tilmicosin solution, the Papp and Ka of the TIL-SLNs obviously increased in the duodenum, but that of the jejunum and ileum showed no change (Supplementary Materials Table S2, Figure 3b, *p* < 0.05). This suggested that prepared TIL-SLNs were satisfactory for our design purpose of achieving duodenum-targeted release and the higher oral absorption of tilmicosin could be expected due to the huge absorption area of the duodenum.

In order to study the transport characteristics of TIL-SLNs and, thus, to achieve the maximum transport efficiency, the duodenum of rat was used as a perfusion section in the subsequent studies. When it was treated with MRP2 (Multidrug resistance proteins-2) inhibitor (indomethacin), the Papp was not obviously changed. After added promotion agent of tight junction (EDTA-2Na), and the P-gp inhibitor (verapamil, polysorbate-80), the Papp was obviously improved (Supplementary Materials Table S3, Figure 3c, *p* < 0.05). The efflux action of TIL-SLNs in the intestinal epithelial cells was related to P-gp rather than MRP2 and the P-gp activity limited the absorption of TIL-SLNs. These reminded us we could combine the promotion agents of tight junction and/or P-gp inhibitors to further improve the oral absorption of tilmicosin enteric granules. Therefore, 1% (*V/W*) polysorbate-80 was added as granulation adhesive for overcoming the efflux of P-gp to TIL-SLNs.

### 3.4. Optimization and Characteristics of Enteric Granules

For preventing the TIL-SLNs from being destroyed by the gastric juice and, thus, avoiding a large amount of premature release before reaching the absorption sites, the enteric granules based on TIL-SLNs were prepared. To ensure the completeness of inner TIL-SLNs of enteric granules, the TIL-SLNs suspension with 5% (*W/V*) sucrose was used as frozen protection during the process of frozen dry by frozen system. Take the costs into consideration, the starch and sucrose were chosen as the filler agent and adhesive of enteric granules. Meanwhile, sucrose was also used as a flavoring agent in the formulation. As mentioned above, the polysorbate-80 could improve the Papp of TIL-SLNs across the duodenum by inhibiting the activation of P-gp. To achieve the maximum absorption of TIL-SLNs in the duodenum, 1% (*V/W*) polysorbate-80 was also added as an adhesive. The completeness of the TIL-SLNs in granules was the most critical issue, which decided whether the TIL-SLNs were present in the enteric granules or not. Therefore, the completeness of the inner TIL-SLNs was further studied in the stability experiments.

**Figure 4:**
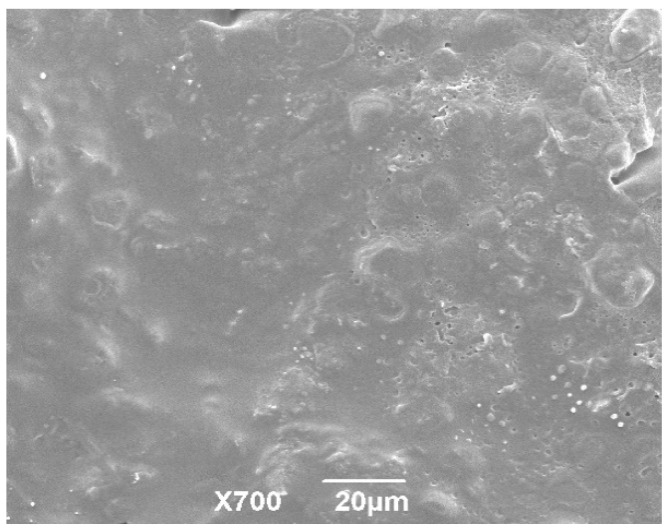
The SEM imaging of the coating membrane of enteric granules. The coating surface showed the relative smooth morphology.

The imaging of the coating membrane indicated that the surface of the granules had a smooth surface morphology (Figure 4). Meanwhile, there were small semi-spherical protrusion and little holes on the surface of the granules. These were possibly caused by solvent evaporation in the coating process [39]. Taking the inconvenient transportation and the storage of the nanosuspension into consideration, many researchers focus on transforming liquid nanoparticles into solid form. Horster et al. [40] prepared a kind of tablet that contained PLGA (poly (lactic-co-glycolic acid)) nanoparticles via a fluid bed granulation. In the industrial production of tilmicosin enteric granules, the spray-dried or fluid-bed technologies can be adopted.

### 3.5. In Vitro Release and Release Mechanisms

**Figure 5:**
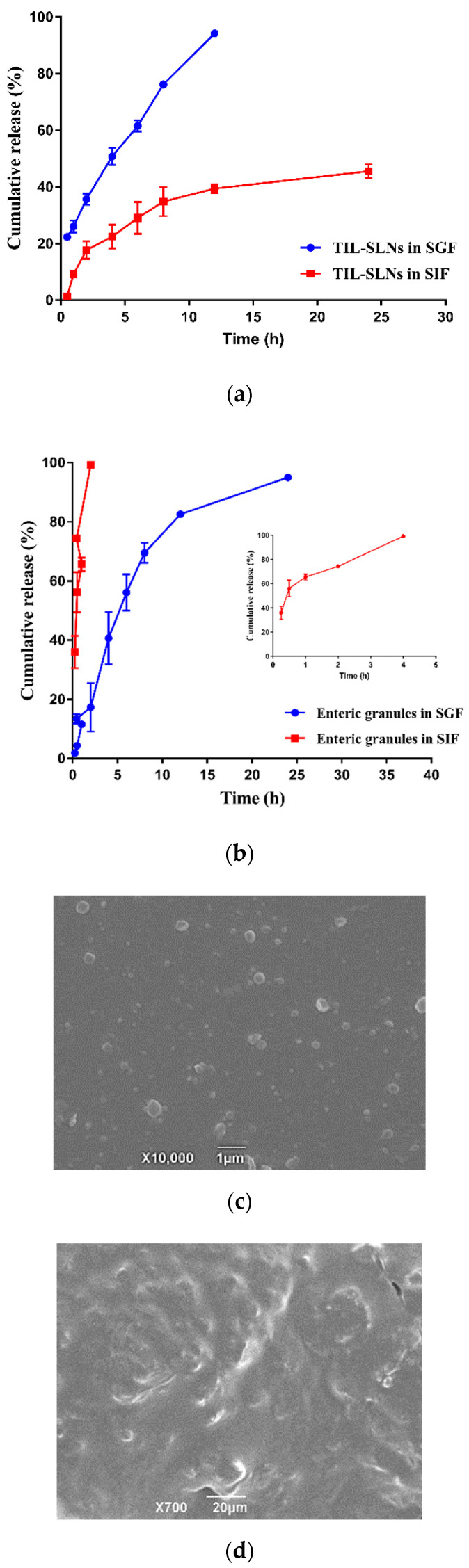

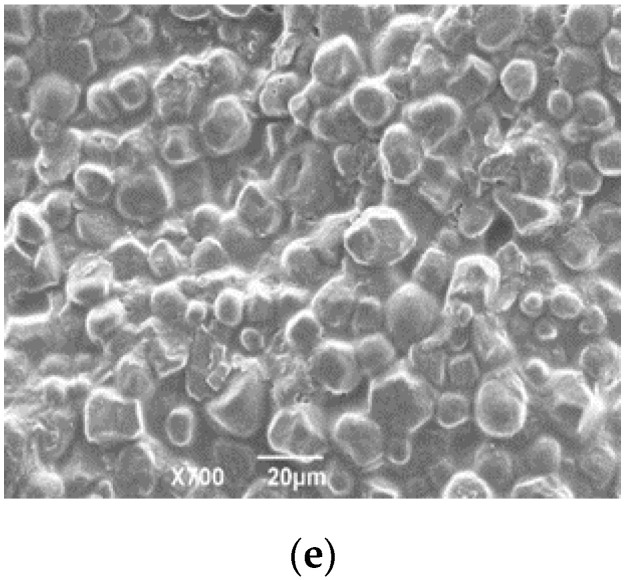
The in vitro release of TIL-SLNs (**a**), the in vitro release of enteric granules (**b**), SEM imaging of the enteric granules when incubated with SIF for 30 min (**c**), and the coting membrane of enteric granules in SGF (**d**) and SIF (**e**) after 30 min incubation. The release rate of TIL-SLNs in SGF was reduced after enteric-coating and the intact TIL-SLNs was observed after the enteric granules were incubated with SIF medium for 30 min.

Compared with the 39.43% tilmicosin in TIL-SLNs released in SIF, the 94.22% tilmicosin in TIL-SLNs was released in SGF within 12 h (Figure 5a). This agrees with the conclusion that the gastric fluid would destroy the TIL-SLNs. As shown in Figure 5b, compared with only 17.34% tilmicosin released in SGF within 2 h, the 99.32% tilmicosin was released in SIF, suggesting that the enteric granules had an obvious faster release rate in SIF than that in SGF. Meanwhile, the intact TIL-SLNs was observed in the SIF medium after the enteric granules were release for 30 min (Figure 5c). Therefore, the enteric granules could deliver more intact TIL-SLNs to the duodenum. Besides, our previous studies indicated that the tilmicosin-loaded hydrogenated castor oil nanoparticles only released 36% within 14 days in the 0.9% (*W/V*) NaCl solution [41]. The too strong sustained-release performance of tilmicosin loaded into the hydrogenated castor oil nanoparticles in neutral would lead to the long-term residence of tilmicosin in intestinal content, and as the intestine transmits, the incomplete released tilmicosin would be discharged from the body, thus resulting in incomplete absorption. Therefore, the carnauba wax that has a higher solubility for tilmicosin and shorter sustained-release performance was selected as a lipid material in this study. Compared to the smooth surface of enteric granules released for 30 min in SGF (Figure 5d), the surface of enteric granules in 30 min SIF was rougher (Figure 5e). Additionally, after being released for 2 h, there were intact granules in the SGF, but no intact granules in the SIF were observed (supplement Figure S2). These demonstrated that the outer enteric coating membrane was dissolved firstly in the process of drug release. After that, the unenveloped tilmicosin was dissolved followed by the disintegration of granules and the inner TIL-SLNs were released. Then, the tilmicosin enveloped in TIL-SLNs was released via erosion and diffusion [20,42,43]. The quicker dissolving of coating membrane and disintegration of enteric granules in SIF more than SGF also supported the conclusion that the enteric granules could deliver more TIL-SLNs to the duodenum.

### 3.6. Stability

**Figure 6:**
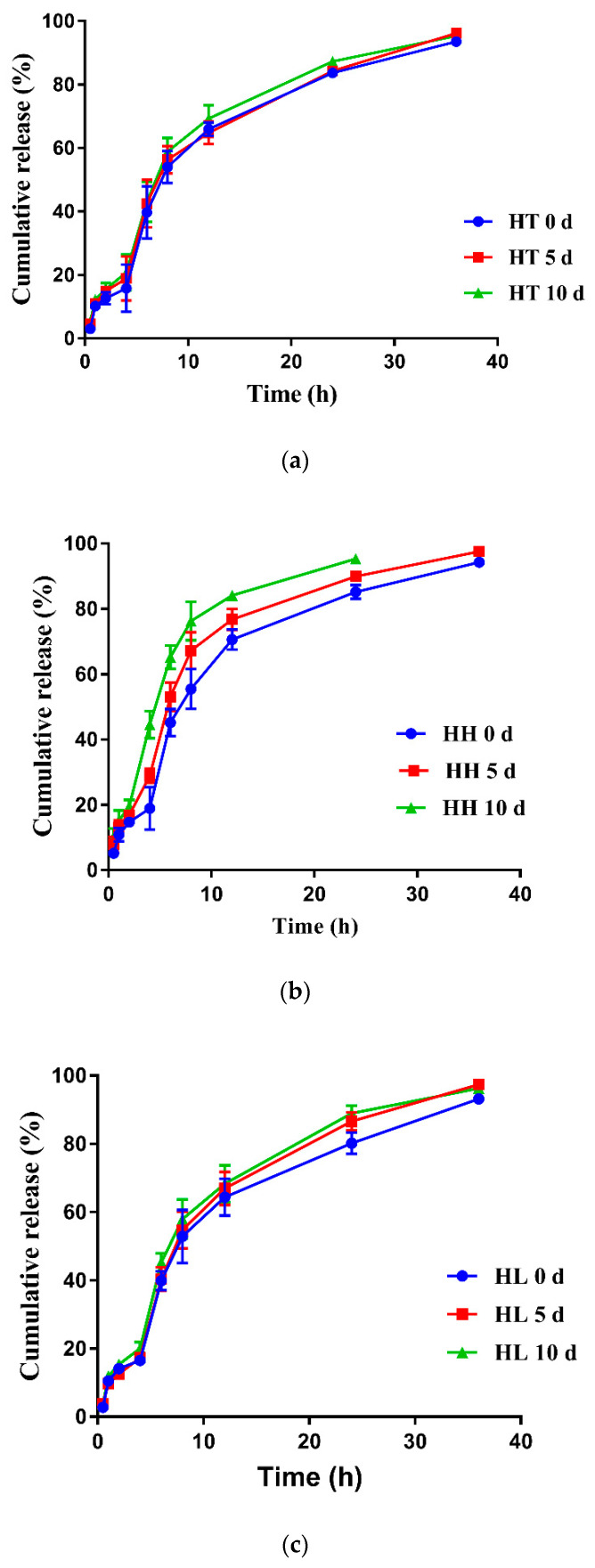
The release profiles of the enteric granules under the influencing factors tests. The release performance of enteric granules showed no obvious change under the influencing factors tests. Note: (**a**): high temperature; (**b**): high humidity; (**c**): high light. HT: high temperature (40 °C); HH: high humidity (25 °C, 90% ± 5%); HL: high light (4500 lux ± 500 lux).

**Figure 7:**
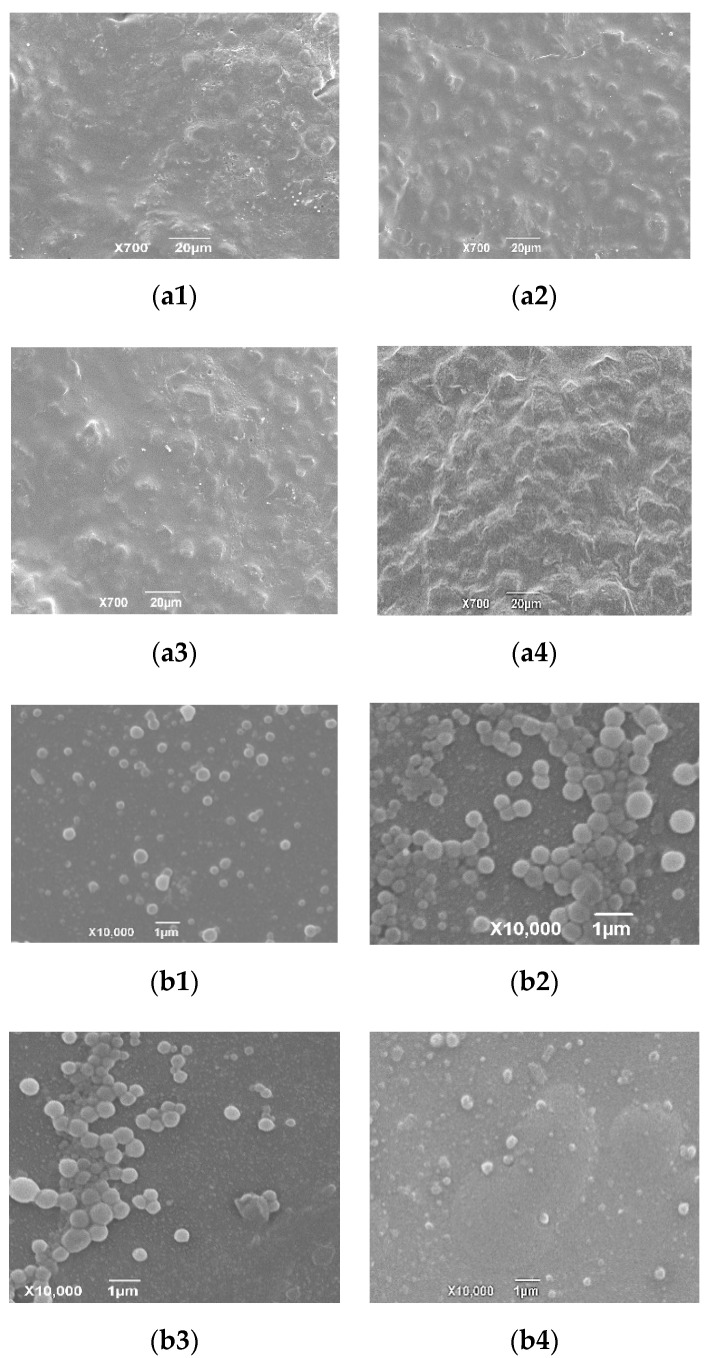
SEM imaging of enteric coating membrane (**a1**–**a4**) and inner TIL-SLNs (**b1**–**b4**) under influence factors experiments. The surface coating membrane and inner TIL-SLNs could keep intact under influence factors experiments. Note: (**a1**): day 0; (**a2**): 10th day under high temperature; (**a3**): 10th day under high humidity; (**a4**): 10th day under high light; (**b1**): day 0; (**b2**): 10th day under high temperature; (**b3**): 10th day under high humidity; (**b4**) 10th day under high light. To verify the completeness of inner TIL-SLNs, the enteric granules under high temperature, high humidity, and high light were ground by mortar and the powder was resuspended in water. Then, the mixture solution was filtered by 0.65 um filter and observed by SEM to verify the completeness of inner TIL-SLNs.

As shown in Table 3, the tilmicosin enteric granules were mildly sensitive to the high temperature of 40 °C. In the high light, 0.36% and 0.59% of the tilmicosin in the enteric granules were degraded after 5 d and 10 d, respectively. In the high humidity, 0.33% and 0.55% of tilmicosin in the granules was degraded after 5 d and 10 d, respectively. The granularity of tilmicosin enteric granules did not have an obvious change. The labeled amount and granularity of the enteric granules agreed with the requirements of Chinese Veterinary Pharmacopoeia 2015 under 10 d influencing factor test. Meanwhile, the release performance of the tilmicosin enteric granules under the high temperature and high light for 10 d showed no obvious change while mild faster release was observed under high humidity for 10 d (Figure 6). These results suggested that the storage stability of the prepared enteric granules could be expected. Additionally, the SEM imaging of coating membrane and TIL-SLNs showed that the completeness of the coating membrane and inner TIL-SLNs would not be changed in the high temperature, high humidity and high light (Figure 7).

### 3.7. Pharmacokinetics

**Figure 8:**
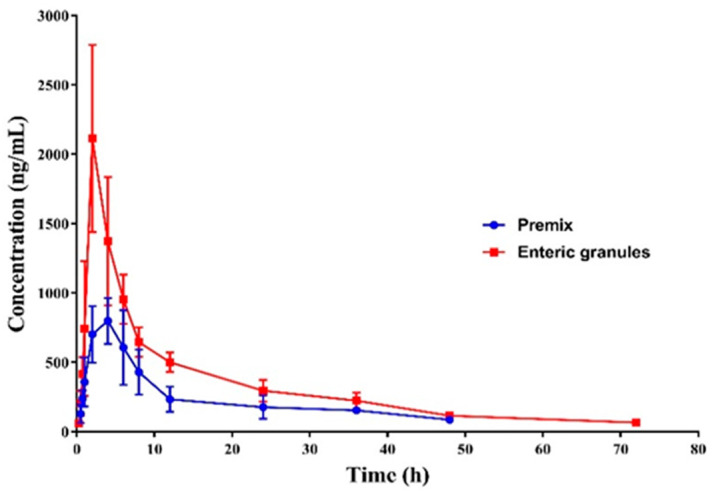
The plasma tilmicosin concentration–time profiles of the prepared enteric granules and commercial premix in pig. The T_max_ of tilmicosin was shortened by 2 h and oral absorption was improved by 2.72 fold by enteric granules.

After administration by the intragastric route, the C_max_ of the commercial tilmicosin premix and the prepared enteric granules were 921 ± 37 ng/mL and 2110 ± 616 ng/mL, respectively, and the time to reach the peak were 4.00 ± 1.26 h and 2 h, respectively. Then, the plasma drug concentration of enteric granules was reduced to 0.07 μg/mL after 72 h. Relatively, the concentration of the commercial premix was swiftly reduced to 0.08 μg/mL within 48 h (Figure 8). The T_1/2β_, mean residence time (MRT) and AUC_0-last_ of the prepared enteric granules were 9.10 ± 3.31 h, 13.13 ± 4.78 h, and 23.17 ± 4.12 μg·h·mL^−1^ respectively, while these parameters for the commercial premix were 7.44 ± 4.02 h, 10.73 ± 5.79 h, and 8.50 ± 3.23 μg·h·mL^−1^, respectively (Table 4). Compared to the commercial premix, the T_1/2β_, MRT, and the oral absorption of the prepared tilmicosin enteric granules were increased by 1.22, 1.22, and 2.72 folds, respectively. Meanwhile, the C_max_, T_max_, and AUC of the TIL-SLNs (same tilmicosin dosage) were 755 ± 55 ng/mL, 2 ± 0 h, and 11.31 µg·h·mL^−1^, respectively (Supplementary Materials Figure S3). The TIL-SLNs showed a lower C_max_ but a larger AUC which suggested a fast release rate of TIL-SLNs in the gastric region and a slow-release rate in the intestines, thus resulting in a lower C_max_ but a higher AUC. Although TIL-SLNs could achieve an earlier T_max_ and a higher AUC, the C_max_ was reduced. Therefore, according to the absorption site of TIL-SLNs (duodenum), it is necessary to reduce its gastric release. After a combination of TIL-SLNs’ technology with enteric coating technology, the enteric granules containing TIL-SLNs achieved an earlier T_max_, satisfactory sustained-release performance, and a higher oral absorption. Combined with the results of in vitro release and intestine perfusion, the higher oral absorption of the enteric granules was attributed to the decreased metabolization of stomach fluid, the duodenum-targeted release, and the highly efficiency of TIL-SLNs across the member of duodenum cells.

In the clinic, tilmicosin is always used to prevent and treat the pneumonia of livestock caused by *Actinobacillus pleuropneumoniae* and *Mycoplasma*. According to previous reports, the minimum inhibitory concentration (MIC) value of tilmicosin against *Actinobacillus pleuropneumoniae* and *Mycoplasma* was 0.5 μg/mL [44] and ≤0.25 μg/mL [45], respectively. From the plasma drug concentration–time curve, we found that not only the C_max_ did not decrease, but the duration of tilmicosin concentration in plasma was higher than the MIC (T > MIC) of *Actinobacillus pleuropneumoniae*, and *Mycoplasma* was also obviously prolonged. Therefore, if the prepared tilmicosin enteric granules was approved to treat *Actinobacillus pleuropneumoniae* and *Mycoplasma* infections of pigs, the dosage frequency of tilmicosin could be reduced. This will help to decrease the consumption of tilmicosin and farming costs. Suggesting the control of duodenum-targeted release and high-efficiency crossmember transport of tilmicosin is an efficient method to reduce the consumption of tilmicosin. For the other VAs, controlling duodenum-targeted release and improving the transport performance to cross the member could reduce consumption, thus decreasing the AMR and environmental exposure risks which would be worth studying. Zhang et al. [46] revealed that enrofloxacin, tilmicosin, ciprofloxacin, and ampicillin are the co-substrate of P-gp and BCRP (Breast cancer resistance protein). The Papp of enrofloxacin, tilmicosin, ciprofloxacin, and ampicillin to the MDCK-chAbcb1 cell models was obviously enhanced by co-incubation with berberine (P-gp inhibitor) [47]. Combining duodenum-targeted release with non-pharmacological activity inhibitor agents will be a potential method to improve the oral absorption of these VAs.

### 3.8. Palatability of Enteric Granules

The average daily feed intake of pigs in various groups was relatively consistent (1.25–1.45 kg/d per group) before the feeding experiment, suggesting the selected pigs were physiological consistent (Table 5). During the feeding experiment (the dose of tilmicosin added was 400 mg/kg feed (FDA)), the average daily feed intake of pigs in the tilmicosin powder group was quickly decreased from about 1.4 kg/d to about 0.9 kg/d. Comparatively, the average daily feed intake of pigs in the enteric granules’ groups showed no significant difference compared to that of the normal daily feed intake and control group. Although the average daily feed intake of pigs in the commercial premix group showed no statistically significant difference (Table 5, *p* < 0.05) compared with the control group, the average daily feed intake in premix group at first three days was obviously lower than that in the control group, which would not be a benefit for the treatment and control of the disease. Feed intake increases in the fourth day in the premix group may be due to a part of bitterness was masked by excipient in premix, and the pigs got used to the bitter taste of tilmicosin after long-time fed. These results indicated that the prepared enteric granules showed more acceptable palatability than commercial tilmicosin premix. It will be a benefit for the clinical application of tilmicosin.

## 4. Conclusions

To provide a kind of tilmicosin product with high oral absorption which can decrease consumption and improve the efficacy of tilmicosin, a duodenum-targeted release and efficient across membrane transport delivery system of tilmicosin was provided in this paper. To control the duodenum targeted absorption, the TIL-SLNs was prepared by the melt emulsification ultrasonic method. The intestine perfusion proved that the TIL-SLNs were mainly absorbed in duodenum. Unfortunately, the intestinal epithelial cells have a strong efflux action against TIL-SLNs transport. On this account, it is a must to overcome this P-gp efflux action; to achieve the maximum duodenum uptake and so the P-gp inhibitor was considered. To reduce the destruction of TIL-SLNs by the gastric fluid, thus achieve the maximum duodenum-targeted release, the enteric coating technology was adopted. Further studies indicated that the prepared tilmicosin enteric granules containing TIL-SLNs could keep intact in SGF for 2 h, suggesting the intact TIL-SLNs could be delivered to the duodenum. Meanwhile, the efflux action of P-gp was inhibited by T-80. Thereby, the duodenum-targeted release and efficient uptake were achieved, and thus the oral absorption of tilmicosin was increased by 2.72 fold. Meanwhile, the feeding experiment demonstrated that the palatability of tilmicosin enteric granules was acceptable for pigs. These results suggest that the enteric granules would reduce the administration frequency and consumption of tilmicosin for pig, thus reduce the costs of farming. This research will provide theoretical directions for the commercial application of nanosuspension and the development of oral products with high oral absorption, thus provide a potential model for reducing the AMR and environmental exposure risks of other VAs.

## Figures and Tables

**Table 1:** Factors and levels of the L9 (3^4^) orthogonal design for SLNs (solid lipid nanoparticles).

Variable	Level		
	1	2	3
Type (A)	PVA (Polyvinyl alcohol)	PVP (Poly-vinyl pyrrolidone)	Poloxamer188
Concentration (B)	1%	2%	3%
Volume (C)	15 mL	20 mL	25 mL

(A) emulsifier type; (B) aqueous phase concentration; (C) aqueous phase volume.

**Table 2:** The optimization of emulsifiers by orthogonal experiment (L_9_3^4^).

Sample	Type (A)	Concentration (B)	Volume (C)	LC (%)	Size (μm)	PDI
1	2	2	3	16.02 ± 0.85	1.34 ± 0.01	>0.50
2	3	1	3	16.52 ± 0.70	1.68 ± 0.01	>0.50
3	2	3	1	18.99 ± 1.97	5.85 ± 0.07	>0.50
4	1	3	3	20.90 ± 0.65	0.34 ± 0.01	0.37 ± 0.02
5	3	3	2	17.61 ± 0.95	5.35 ± 0.04	>0.50
6	3	2	1	17.04 ± 1.65	2.45 ± 0.01	>0.50
7	1	1	1	17.19 ± 0.98	0.48 ± 0.01	0.42 ± 0.03
8	1	2	2	15.47 ± 0.98	0.31 ± 0.01	0.31 ± 0.02
9	2	1	2	15.91 ± 1.23	1.42 ± 0.01	>0.50
k1	17.85	16.54	17.74			
k2	16.64	15.84	16.33			
k3	17.05	19.16	17.81			
R	1.21	3.32	1.48			
Optimum	A1	B3	C3			

Notes: k_1_, k_2_, and k_3_ are the average of grade for 3 levels in each factor; R is the difference value between the max and mix of k_1_, k_2_, and k_3_ in each level. Abbreviations: LC: load capacity; PDI: poly dispersion index.

**Table 3:** The influence factor test of tilmicosin enteric granules (Mean ± SD, *n* = 3).

Time	Factors	Appearance	Content (%)	Unqualified Granularity (%)
0th d	High temperature	Yellowish	9.95 ± 0.14	8.28 ± 0.77
	High humidity	Yellowish	9.95 ± 0.24	8.28 ± 0.77
	High light	Yellowish	9.95 ± 0.15	8.28 ± 0.77
5th d	High temperature	Yellowish	9.40 ± 0.14	8.02 ± 0.52
	High humidity	Yellowish	9.62 ± 0.21	8.45 ± 0.64
	High light	Yellowish	9.59 ± 0.18	8.18 ± 0.89
10th d	High temperature	Yellowish	9.24 ± 0.13	7.94 ± 0.43
	High humidity	Yellowish	9.44 ± 0.24	8.89 ± 1.01
	High light	Yellowish	9.36 ± 0.10	8.05 ± 0.61

High temperature: 60 °C; High humidity: 25 °C; and 90% ± 5%; High light: 4500 lux ± 500 lux.

**Table 4:** Pharmacokinetic parameters of tilmicosin after oral administration of tilmicosin granules and premix (Mean ± SD, *n* = 6).

Parameter	Unit	Enteric Granules	Premix
Vz F	mL/kg	234,913.52 ± 50216.91	212,708.7 ± 54,024.98
MRT	h	13.13 ± 4.78	10.73 ± 5.79
AUC_0-last_	µg·h·mL^−1^	23.17 ± 4.12	8.50 ± 3.23
T_1/2β_	h	9.10 ± 3.31	7.44 ± 4.02
CL	L/h/kg	8203.84 ± 1374.71	23,895.02 ± 8991.91
T_max_	h	2 ± 0	4.0 ± 1.26
C_max_	µg/mL	2.11 ± 0.62	0.92 ± 0.04
F (%)	/	272.46%	/

AUC_0–last_: area under the curve; CL: body clearance; T_1/2β_: elimination half-life; MRT: mean residence time; C_max_: peak concentration; T_max_: the time reach to the peak concentration; F: relative bioavailability.

**Table 5:** The daily feed intake of pigs (Mean ± SD, *n* = 3).

Groups	Before Experiment (kg/group)				During Experiment (kg/group)					
	1d	2d	3d	x ± SD	1d	2d	3d	4d	5d	x ± SD
Control	1.30	1.28	1.35	1.32 ± 0.02	1.35	1.32	1.40	1.38	1.43	1.38 ± 0.04
Mixed Tilmicosin	1.24	1.22	1.28	1.25 ± 0.02	0.95	0.96	0.88	0.97	0.77	0.91 ± 0.07^a^
Premix	1.26	1.20	1.23	1.23 ± 0.02	0.74	0.80	1.05	1.38	1.46	1.09 ± 0.29
Enteric Granules	1.39	1.33	1.35	1.36 ± 0.02	1.10	1.25	1.32	1.59	1.58	1.37 ± 0.19^b^

Control group was addition feed only; Mixed tilmicosin: Feed was added native tilmicosin; Premix: The commercial tilmicosin premix. ^a^ Statistically significant compared with control; ^b^ Statistically significant compared with mixed tilmicosin. The statistical difference was analyzed by one-way analysis of variance at *p* < 0.05.

## Supplementary Materials

The following are available online at https://www.mdpi.com/1999-4923/12/8/731/s1, Table S1: Papp and Ka of various concentration TIL-SLNs in duodenum (Mean ± SD, n = 3), Table S2: Papp and Ka of TIL-SLNs and solution in various intestine sections (Mean ± SD, n = 3), Table S3: The influence of various inhibitors to Papp and Ka of TIL-SLNs (Mean ± SD, n = 3), Figure S1: The DSC thermograms of pure tilmicosin, carnauba wax and TIL-SLNs, Figure S2: The in vitro release imaging of 2h SGF (a) and SIF (b) medium, Figure S3: The plasma tilmicosin concentration-time profiles of the TIL-SLNs.

## References

[1] Mole B. (2013). MRSA: Farming up trouble. Nature.

[2] Ferri M., Ranucci E., Romagnoli P., Giaccone V. (2017). Antimicrobial resistance: A global emerging threat to public health systems. Crit. Rev. Food Sci. Nutr..

[3] Bauer K.A., Kullar R., Gilchrist M., File T.M. (2019). Antibiotics and adverse events: The role of antimicrobial stewardship programs in doing no harm. Curr. Opin. Infect. Dis..

[4] Choonara B.F., Choonara Y.E., Kumar P., Bijukumar D., Toit L.D., Pillay V. (2014). A review of advanced oral drug delivery technologies facilitating the protection and absorption of protein and peptide molecules. BioTechnol. Adv..

[5] Rani S., Rana R., Saraogi G.K., Kumar V., Gupta U. (2019). Self-emulsifying oral lipid drug delivery systems: Advances and challenges. AAPS PharmSciTech.

[6] Raza A., Sime F.B., Cabot P.J., Maqbool F., Maqbool F., Roberts J.A., Falconer J.R. (2019). Solid nanoparticles for oral antimicrobial drug delivery: A review. Drug Discov. Today.

[7] Sarmah A.K., Meyer M.T., Boxall A.B. (2006). A global perspective on the use, sales, exposure pathways, occurrence, fate and effects of veterinary antibiotics (VAs) in the environment. Chemosphere.

[8] Xie S.Y., Tao Y.F., Pan Y.H., Qu W., Cheng G.Y., Huang L.L., Yuan Z.H. (2014). Biodegradable nanoparticles for intracellular delivery of antimicrobial agents. J. Control. Release.

[9] Wang F., Cao J., Hao J., Liu K. (2014). Pharmacokinetics, tissue distribution and relative bioavailability of geniposide-solid lipid nanoparticles following oral administration. J. Microencapsul..

[10] Du Y., Ling L., Ismail M., He W., Xia Q. (2018). Redox sensitive lipid-camptothecin conjugate encapsulated solid lipid nanoparticles for oral delivery. Int. J. Pharm..

[11] Tao Y.F., Yang F., Meng K., Zhou K., Luo W., Qu W., Pan Y., Yuan Z., Xie S. (2019). Exploitation of enrofloxacin-loaded docosanoic acid solid lipid nanoparticle suspension as oral and intramuscular sustained release formulations for pig. Drug Deliv..

[12] Patel M.H., Mundada V.P., Sawant K.K. (2019). Fabrication of solid lipid nanoparticles of lurasidone HCl for oral delivery: Optimization, in vitro characterization, cell line studies and in vivo efficacy in schizophrenia. Drug Dev. Ind. Pharm..

[13] Ali H., Singh S.K. (2016). Biological Voyage of solid lipid nanoparticles: A proficient carrier in nanomedicine. Ther. Deliv..

[14] Li H., Zhao X., Ma Y., Zhai G., Li L., Lou H. (2009). Enhancement of gastrointestinal absorption of quercetin by solid lipid nanoparticles. J. Control. Release.

[15] Zhang N., Ping Q., Huang G., Han X., Cheng Y., Xu W. (2006). Transport characteristics of wheat germ agglutinin-modified insulin-liposomes and solid lipid nanoparticles in a perfused rat intestinal model. J. Nanosci. Nanotechnol..

[16] Carmona-Ibáñez G., del Val Bermejo-Sanz M., Rius-Alarcó F., Martín-Villodre A. (1999). Experimental studies on the influence of surfactants on intestinal absorption of drugs. Cefadroxil as model drug and sodium taurocholate as natural model surfactant: Studies in rat colon and in rat duodenum. Arzneimittelforschung.

[17] Zhang Z., Gao F., Bu H., Xiao J., Li Y. (2012). Solid lipid nanoparticles loading candesartan cilexetil enhance oral bioavailability: In vitro characteristics and absorption mechanism in rats. Nanomedicine.

[18] Wang J., Li L., Wu L., Du Y., Sun J., Wang Y., Fu Q., Zhang P., He Z. (2017). Development of novel self-assembled ES-PLGA hybrid nanoparticles for improving oral absorption of doxorubicin hydrochloride by P-gp inhibition: In vitro and in vivo evaluation. Eur. J. Pharm. Sci..

[19] Cornaire G., Woodley J., Hermann P., Cloarec A., Arellano C., Houin G. (2004). Impact of excipients on the absorption of P-glycoprotein substrates in vitro and in vivo. Int. J. Pharm..

[20] Li C., Zhou K., Chen D., Xu W., Tao Y., Pan Y., Meng K., Shabbir M.A.B., Liu Q., Huang L. (2019). Solid lipid nanoparticles with enteric coating for improving stability, palatability, and oral bioavailability of enrofloxacin. Int. J. Nanomed..

[21] Bargoni A., Cavalli R., Caputo O., Fundarò A., Gasco M.R., Zara G.P. (1998). Solid lipid nanoparticles in lymph and plasma after duodenal administration to rats. Pharm. Res..

[22] Cavalli R., Bargoni A., Podio V., Muntoni E., Zara G.P., Gasco M.R. (2003). Duodenal administration of solid lipid nanoparticles loaded with different percentages of tobramycin. J. Pharm. Sci..

[23] Manjunath K., Venkateswarlu V. (2005). Pharmacokinetics, tissue distribution and bioavailability of clozapine solid lipid nanoparticles after intravenous and intraduodenal administration. J. Control. Release.

[24] Jordan W.H., Byrd R.A., Cochrane R.L., Hanasono G.K., Hoyt J.A., Main B.W., Meyerhoff R.D., Sarazan R.D. (1993). A review of the toxicology of the antibiotic MICOTIL 300. Vet. Hum. Toxicol..

[25] Ramadan A. (1997). Pharmacokinetics of tilmicosin in serum and milk of goats. Res. Vet. Sci..

[26] Abdel-Daim M.M., Ghazy E.W., Fayez M. (2015). Synergistic protective role of mirazid (commiphora molmol) and ascorbic acid against tilmicosin-induced cardiotoxicity in mice. Can. J. Physiol. Pharmaco..

[27] Li B., Gong S.Y., Zhou X.Z., Yang Y.J., Li J.Y., Wei X.J., Cheng F.S., Niu J.R., Liu X.W., Zhang J.Y. (2017). Determination of antibacterial agent tilmicosin in pig plasma by LC/MS/MS and its application to pharmacokinetics. Biomed. Chromatogr..

[28] Zhang P., Hao H., Li J., Cheng G., Chen D., Tao Y., Huang L., Wang Y., Dai M., Liu Z. (2016). The epidemiologic and pharmacodynamic cutoff values of tilmicosin against haemophilus parasui. Front. Microbiol..

[29] Lim J., Yun H. (2001). Postantibiotic effects and postantibiotic sub-mic effects of erythromycin, roxithromycin, tilmicosin, and tylosin on pasteurella multocida. Int. J. Antimicrob. Agents.

[30] Zhou K., Wang X., Chen D., Yuan Y., Wang S., Li C., Yan Y., Liu Q., Shao L., Huang L. (2019). Enhanced treatment effects of tilmicosin against staphylococcus aureus cow mastitis by self-assembly sodium alginate-chitosan nanogel. Pharmaceutics.

[31] Yang H., Zhai B., Fan Y., Wang J., Sun J., Shi Y., Guo D. (2018). Intestinal absorption mechanisms of araloside A in situ single-pass intestinal perfusion and in vitro Caco-2 cell model. Biomed. Pharmacother..

[32] Chen S., Zhang J., Wu L., Wu H., Dai M. (2018). Paeonol nanoemulsion for enhanced oral bioavailability: Optimization and mechanism. Nanomedicine.

[33] Dubbelboer I.R., Dahlgren D., Sjögren E., Lennernäs H. (2019). Rat intestinal drug permeability: A status report and summary of repeated determinations. Eur. J. Pharm. Biopharm..

[34] Dahlgren D., Roos C., Peters K., Lundqvist A., Tannergren C., Sjögren E., Sjöblom M., Lennernäs H. (2019). Evaluation of drug permeability calculation based on luminal disappearance and plasma appearance in the rat single-pass intestinal perfusion model. Eur. J. Pharm. Biopharm..

[35] Zhang B., Lu Y., Li P., Wen X., Yang J. (2019). Study on the absorption of corosolic acid in the gastrointestinal tract and its metabolites in rats. Toxicol. Appl. Pharmacol..

[36] He X., Song Z.J., Jiang C.P., Zhang C.F. (2017). Absorption properties of luteolin and apigenin in genkwa flos using in situ single-pass intestinal perfusion system in the rat. Am. J. Chin. Med..

[37] Medarević D.P., Kachrimanis K., Mitrić M., Djuriš J., Djurić Z., Ibrić S. (2016). Dissolution rate enhancement and physicochemical characterization of carbamazepine-poloxamer solid dispersions. Pharm. Dev. Technol..

[38] Hu X.Y., Lou H., Hageman M.J. (2018). Preparation of lapatinib ditosylate solid dispersions using solvent rotary evaporation and hot melt extrusion for solubility and dissolution enhancement. Int. J. Pharm..

[39] Ali S.F.B., Afrooz H., Hampel R., Mohamed E.M., Bhattacharya R., Cook P., Khan M.A., Rahman Z. (2019). Blend of cellulose ester and enteric polymers for delayed and enteric coating of core tablets of hydrophilic and hydrophobic drugs. Int. J. Pharm..

[40] Horster L., Bernhardt A., Kiehm K., Langer K. (2019). Conversion of PLGA nanoparticle suspensions into solid dosage forms via fluid bed granulation and tableting. Eur. J. Pharm. Biopharm..

[41] Wang X.F., Zhang S.L., Zhu L.Y., Xie S.Y., Dong Z., Wang Y., Zhou W.Z. (2012). Enhancement of antibacterial activity of tilmicosin against Staphylococcus aureus by solid lipid nanoparticles in vitro and in vivo. Vet. J..

[42] zur Mühlen A., Schwarz C., Mehnert W. (1998). Solid lipid nanoparticles (SLN) for controlled drug delivery--drug release and release mechanism. Eur. J. Pharm. Biopharm..

[43] Müller R.H., Mäder K., Gohla S. (2000). Solid lipid nanoparticles (SLN) for controlled drug delivery-a review of the state of the art. Eur. J. Pharm. Biopharm..

[44] Ling Z., Yonghong L., Junfeng L., Li Z., Xianqiang L. (2018). Tilmicosin- and florfenicol-loaded hydrogenated castor oil-solid lipid nanoparticles to pigs: Combined antibacterial activities and pharmacokinetics. J. Vet. Pharmacol. Ther..

[45] Kreizinger Z., Grózner D., Sulyok K.M., Nilsson K., Hrivnák V., Benčina D., Gyuranecz M. (2017). Antibiotic susceptibility profiles of mycoplasma synoviae strains originating from central and eastern Europe. BMC Vet. Res..

[46] Zhang Y., Huang J., Liu Y., Guo T., Wang L. (2018). Using the lentiviral vector system to stably express chicken P-gp and BCRP in MDCK cells for screening the substrates and studying the interplay of both transporters. Arch. Toxicol..

[47] Zhang Y., Guo L., Huang J., Sun Y., He F., Zloh M., Wang L. (2019). Inhibitory Effect of Berberine on Broiler P-glycoprotein Expression and Function: In Situ and In Vitro Studies. Int. J. Mol. Sci..

