# Supplementary material for: Solid Lipid Nanoparticles for Duodenum Targeted Oral Delivery of Tilmicosin
Source: Pharmaceutics. 2020 Aug 4;12(8):731. doi: 10.3390/pharmaceutics12080731 (PMC7466129; doi:10.3390/pharmaceutics12080731)
Supplement: Supplementary file 1 [file pharmaceutics-12-00731-s001.pdf]

pharmaceutics

Supplementary Materials: Solid Lipid Nanoparticles for Duodenum Targeted Oral Delivery of Tilmicosin

Kaixiang Zhou, Yuanyuan Yan, Dongmei Chen, Lingli Huang, Chao Li, Kuiyu Meng, Shuge Wang, Samah Attia Algharib, Zonghui Yuan and Shuyu Xie

Table S1. Papp and Ka of various concentration TIL-SLNs in duodenum (Mean ± SD, n = 3).

Concentration (mg/mL)	Papp (×10^{-3} cm/min)	Ka (×10^{-3}/min)
0.06	0.63 ± 0.05	2.13 ± 0.18
0.125	0.77 ± 0.05	1.97 ± 0.20
0.5	0.70 ± 0.14	1.84 ± 0.36

Table S2. Papp and Ka of TIL-SLNs and solution in various intestine sections (Mean ± SD, n = 3).

Preparations	Intestine section	Papp (×10^{-3}cm/min)	Ka (×10^{-2}min^{-1})
TIL-SLNs	Duodenum	0.77 ± 0.05	1.97 ± 0.20
	Jejunum	0.09 ± 0.04^{a}	0.25 ± 0.05
	Ileum	0.60 ± 0.16^{b}	1.73 ± 0.08
Solution	Duodenum	0.37 ± 0.09^{abc}	1.18 ± 0.22
	Jejunum	0.09 ± 0.02^{acd}	0.20 ± 0.09
	Ileum	0.67 ± 0.09^{bde}	1.90 ± 0.07

Note: [a]: Statistically significances from TIL-SLNs duodenum; [b]: Statistically significances form TIL-SLNs jejunum; [c]: Statistically significances from TIL-SLNs ileum; [d]: Statistically significances from solution duodenum; [e]: Statistically significances from solution jejunum. The statistical difference was analyzed by one-way analysis of variance at $p < 0.05$.

Table S3. The influence of various inhibitors to Papp and Ka of TIL-SLNs (Mean ± SD, n = 3).

Inhibitors	Papp (×10^{-3} cm/min)	Ka (×10^{-2}/min)
Pure TIL-SLNs (0.125 mg/mL)	0.77±0.05	1.97±0.20
Verapamil	4.20±0.06^{a}	8.05±0.33
Indomethacin	1.03±0.19^{ab}	2.60±0.42
EDTA-2Na	2.89±0.25^{abc}	6.18±0.41
0.5% Tween 80	1.27±0.05^{abd}	3.25±0.44
1% Tween 80	1.79±0.25^{abcde}	4.20±0.51

Note: [a]: Statistically significances from pure TIL-SLNs (0.125 mg/mL); [b]: Statistically significances form verapamil; [c]: Statistically significances from indomethacin; [d]: Statistically significances from EDTA-2Na; [e]: Statistically significances from 0.5% T-80. The statistical difference was analyzed by one-way analysis of variance at $p < 0.05$.

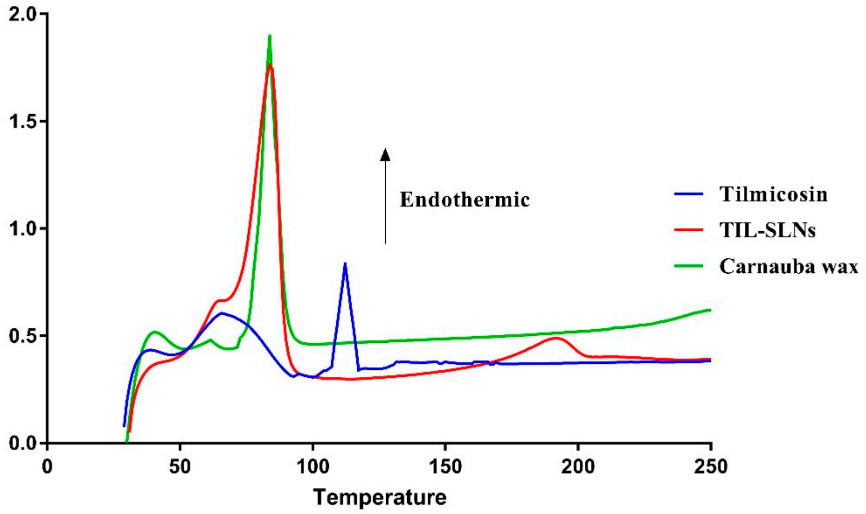

Figure S1. The DSC thermograms of pure tilmicosin, carnauba wax and TIL-SLNs.

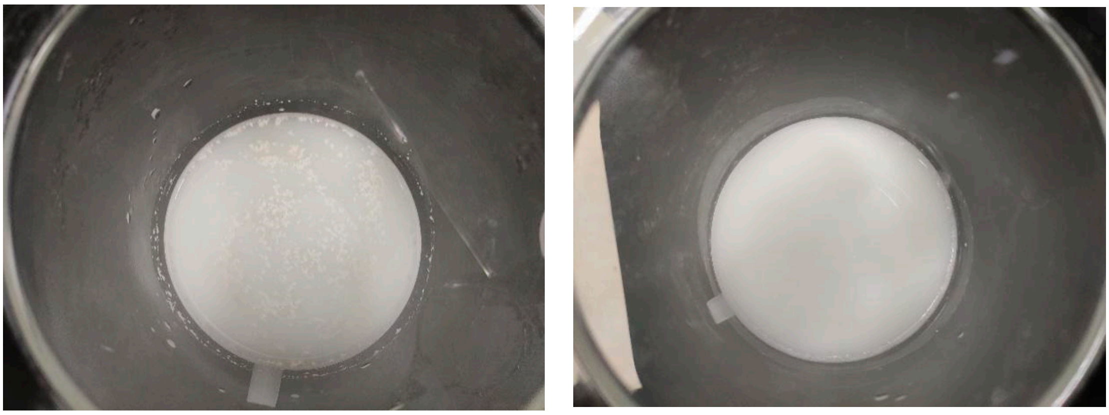

(a) (b)

Figure S2. The in vitro release imaging of 2h SGF (a) and SIF (b) medium.

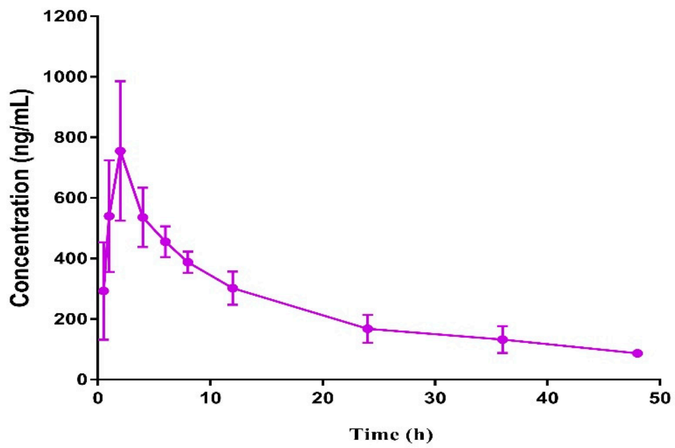

Figure S3. The plasma tilmicosin concentration-time profiles of the TIL-SLNs. Note: Key parameters**:** area under the curve (AUC): 11.31 μg·h·mL^{-1}, peak concentration (C_{max}): 755 ± 55 ng/mL, time reach to the peak concentration (T_{max}): 2 h.
